# Self-care and health seeking for diabetes and hypertension in Cambodia

**DOI:** 10.1093/heapol/czaf039

**Published:** 2025-06-17

**Authors:** Marius Wamsiedel, Dyna Khuon, Yunguo Liu, Vonthanak Saphonn

**Affiliations:** Global Health Research Center, Duke Kunshan University, 8, Duke Avenue, Kunshan, Jiangsu Province, China; University of Health Sciences, 73 Preah Monivong Blvd (93), Phnom Penh, Cambodia; Global Health Research Center, Duke Kunshan University, 8, Duke Avenue, Kunshan, Jiangsu Province, China; University of Health Sciences, 73 Preah Monivong Blvd (93), Phnom Penh, Cambodia

**Keywords:** non-communicable diseases, diabetes, hypertension, medical pluralism, health-seeking behavior, village doctors, Health Equity Fund, National Social Security Fund

## Abstract

Cambodia is experiencing a growing burden of non-communicable diseases (NCDs) as it undergoes an epidemiological transition. This qualitative study investigates the health-seeking behaviors of Cambodians in the context of hypertension and diabetes, focusing on the utilization of both formal healthcare and alternative medical practices. Data are from 20 in-depth interviews with participants without social health protection and 6 focus groups (*n* = 48), involving beneficiaries of the Health Equity Fund (HEF) and National Social Security Fund (NSSF). The research explores personal experiences with NCD management, perceptions of social health protection schemes, and perceived barriers to accessing healthcare. Data were collected in urban and rural settings in Cambodia, with thematic analysis facilitated by NVivo 14 software. Many participants delayed seeking biomedical advice due to economic constraints, cultural beliefs, and perceived inadequacies in the healthcare system. Traditional remedies and self-medication were commonly reported, often due to their accessibility and lower cost compared to biomedical healthcare services. Despite the availability of HEF and NSSF, structural challenges within the healthcare system, such as shortages of medications and trained staff at public health centers, emerged as significant barriers. Pharmacy workers and village healers are insufficiently utilized human resources. Formalizing their role in the secondary prevention of NCDs could contribute to the early detection of diabetes and hypertension. The findings suggest the need for an integrated health system that strengthens the capacity of primary care facilities to manage NCDs effectively and utilizes the semi-professional sector more systematically. Strengthening primary care, expanding service availability, and improving social health protection schemes are essential to reduce health disparities and improve access to quality care for NCDs in Cambodia.

Key messages

- Participants tend to recognize hypertension and diabetes as lifestyle-related and increasingly prevalent conditions, but many hold misconceptions about their risk factors and prevention.
- Secondary prevention of hypertension and diabetes is affected by the insufficient awareness of screenings, fear of diagnosis, financial barriers to biomedical care, and the lack of diabetes screening at many health centers.
- Although the expansion of social health protection schemes (Health Equity Fund and National Social Security Fund) is important for the management of non-communicable diseases, its impact is limited by the frequent medicine shortages and long waiting times in primary healthcare settings.
- Leveraging the semi-professional sector of care, by providing training and equipment to pharmacy workers and village healers, could improve the early detection of diabetes and hypertension.

## Introduction

Like other developing economies undergoing epidemiological transition, Cambodia currently faces the double burden of communicable and non-communicable diseases (NCDs). Cardiovascular diseases, diabetes, chronic respiratory diseases, and cancer claimed 65 400 lives in 2019, accounting for over two-thirds (68%) of all deaths in the country (World Health Organization 2022). In contrast, NCDs caused only 39% of the total mortality in Cambodia in 2000 (World Bank). The estimated number of people living with diabetes increased 3-fold between 2011 and 2021, reaching almost 600 000 individuals (International Diabetes Federation 2021). Moreover, ∼45% of the people living with diabetes have not been diagnosed (Te *et al.* 2023). The prevalence of hypertension in Cambodia was estimated at 11.2% in 2010 for the age group 25–64 years (University of Health Sciences 2010) and 14.2% in 2016 for those aged 18–69 years (Chhim *et al.* 2023). The prevalence rises with age, with 23.5% of people aged 40–69 years being hypertensive in 2016 (Chham *et al.* 2022). The prevalence of both diabetes and hypertension is expected to continue increasing in the coming years due to economic development, urbanization, and lifestyle changes.

The health authorities in Cambodia have recognized the need to strengthen the health system's response to the rising prevalence of NCDs. In 2014, one of the steps taken in this direction was the adoption of a standard operating procedure for managing hypertension and diabetes in primary care [Ministry of Health (Cambodia) 2019], inspired by the WHO Package of Essential Noncommunicable Disease Interventions for Primary Health Care (World Health Organization 2010). Its aim was to involve health centers in the prevention, early detection, and comprehensive management of these two NCDs in order to reduce the disease burden and its associated costs. However, a systematic survey conducted in 2020 revealed that only 19% of the country's health centers provided diabetes services, 20% had at least one staff member trained in diabetes care, and 23% had anti-diabetic medicines available (Te *et al.* 2022). The lack of adequate staff and medicines in health centers, coupled with prolonged waiting times and uncertain outcomes, undermines trust in primary care (Chham *et al.* 2023) and drives people to seek alternatives.

The options available are many. In Cambodia, medical pluralism allows for a range of healthcare providers, including qualified physicians who often engage in dual practice across the public and private sectors (Khun and Manderson 2007); village healers (pet phum) who have acquired limited, if any, biomedical training but have a certain experiential knowledge that confers on them credibility and acceptability in the poorly serviced rural areas (Gryseels *et al.* 2019); and indigenous healers, including spiritual ones (Ovesen and Trankell 2016). In addition, many pharmacies and drugstores not only sell medicines but also provide informal diagnoses and treatments that are consistent with local health beliefs and expectations (Gryseels *et al.* 2019). These establishments, staffed by licensed and unlicensed pharmacists, are particularly popular for treating conditions that are perceived as minor (Ozawa and Walker 2011), despite the potential risks associated with such practices.

Timely medical care and adherence to treatment are essential for effectively managing hypertension and diabetes. However, previous studies have indicated that people in Cambodia often delay seeking medical care for economic, cultural, and social reasons. The cost of examinations, laboratory tests, and treatment in both public and private sectors, compounded by indirect costs such as transportation, occasional informal payments, and the productive time lost when visiting the doctor, deter many from seeking care even when experiencing concerning symptoms. Folk health beliefs, such as supernatural disease causation—where health deterioration is believed to result from moral transgressions that attract the ire of ancestors’ spirits or deities—also lead many people to spiritual healing (Grundy *et al.* 2016). Others turn to traditional Khmer remedies due to concerns over biomedicine's healing potential or side effects (Gryseels *et al.* 2019), lack of trust in the competence or professionalism of licensed doctors (Ros *et al.* 2018), dissatisfaction with treatment in the public sector (Res 2017, Verschuere *et al.* 2017), and the costs in the private sector. Lack of awareness about the symptoms and risk factors associated with diabetes and hypertension has also been identified as a determinant of delays in seeking care (Chhim *et al.* 2023), although one study found that participants were generally knowledgeable about NCDs and their management (Steinman *et al.* 2020). Since family plays an important role in shaping decision-making related to health (Ovesen and Trankell 2016), it is sometimes a contributor to the delays in seeking care. Even when they resort to biomedical professionals, people living with diabetes and hypertension are sometimes not diagnosed due to the unavailability of screening and testing at many healthcare facilities (Jacobs *et al.* 2017).

A systematic review conducted a decade and a half ago (Grundy and Annear 2010) noted the scarcity of studies on pathways of care in Cambodia. Since then, the situation has improved, with qualitative studies examining health-seeking behavior for a variety of conditions, including enteric fever (Kuijpers *et al.* 2018), malaria (Verschuere *et al.* 2017), tuberculosis (Teo *et al.* 2020), and diabetes (Jacobs *et al.* 2017, Nang *et al.* 2019). This study extends the discussion by examining how people's decision-making in dealing with symptoms associated with hypertension and diabetes is influenced by the recent transformation of the health system.

To increase access to quality healthcare while reducing out-of-pocket payments and the risk of catastrophic health expenditure, Cambodia has committed to universal health coverage and has developed two social health protection schemes: the Health Equity Fund (HEF) and the National Social Security Fund (NSSF), which exclusively cover allopathic medical care. Initiated as a pilot project by the international non-governmental organization (NGO) ‘Doctors Without Borders’ in 2000 (Noirhomme *et al.* 2007), HEF was institutionalized as a national program 15 years later (Kolesar *et al.* 2021b). This scheme targets the most economically deprived members of society, who are identified through the periodic PoorID surveys conducted by the Ministry of Planning or directly at healthcare facilities. Since late 2017, it has also extended coverage to various categories of people who do not meet the poverty criterion, including village chiefs and deputy chiefs, commune council members, and professional sports players (Kolesar). HEF covers not only medical costs but also indirect expenses (e.g. transportation, meals) that prevent many low-income people from seeking care. However, this scheme is restricted to the public sector of care (Kolesar *et al.* 2021a), despite private facilities being the most attractive option for seeking care in the country (Khun and Manderson 2007, Ozawa and Walker 2011). Health facilities are reimbursed directly, without the need for out-of-pocket payments on the part of the beneficiary.

NSSF covers civil servants, retirees, and veterans (NSSF-C) as well as formally employed workers (NSSF-F). Administered by the Ministry of Labor and Vocational Training, it serves >2 million persons, not including their dependents (Kolesar *et al.* 2021b). Although the entitlement to services is the same for the two sub-schemes, the contribution rate is higher in the NSSF-F scheme compared to NSSF-C (2.6% vs. 1%, respectively), which raises equity concerns (Kwon and Keo 2019). The scheme can be used in the public sector as well as in contracted private health facilities.

This paper examines how people navigate a pluralistic health system undergoing radical changes when experiencing symptoms typically associated with hypertension and diabetes, and how they manage their NCD once diagnosed. At the same time, it looks for feasible solutions to improve the detection and management of NCDs in Cambodia.

## Methods

This paper draws upon 20 in-depth interviews (IDIs) with people without any social health protection and 6 focus groups (FGs, *n* = 48) with HEF or NSSF beneficiaries. The individual interviews generated insights into the challenges experienced by uninsured people in dealing with health issues, whereas FGs were chosen to understand how people collectively make sense of the advantages and limitations of social health protection schemes and unravel the social expectations regarding seeking health (see Table 1).

**Table 1.** Age and gender structure of the participants in the in-depth interviews.

Age group, years	Female	Male	Total
18–25	1	0	1
26–35	2	0	2
36–45	3	3	6
46–55	1	1	2
56–65	2	3	5
66+	1	1	2
Not specified	2	0	2
Total	12	8	20

The interview guide was semi-structured, which ensured that the issues of interest were covered systematically while participants had the chance to steer the conversation to what they regarded as being relevant. The guide covered the participants’ perception of diabetes and high blood pressure, their health-seeking behavior and experiences, and their positioning towards social health protection schemes, particularly in relation to NCDs. To stimulate a discussion of the perceived barriers to seeking care and the potential consequences of delayed health-seeking, participants were invited to discuss a hypothetical scenario involving a person who suspects they have high blood pressure but does not go for a check-up. The FG guide complemented the interview guide, but was designed in a way that encouraged participants to make sense together, through three hypothetical scenarios, of the challenges associated with the prevention, diagnosis, and management of diabetes and hypertension. Fieldwork was conducted in two rounds, in June and October 2023, in three locations: the capital city (Phnom Penh), its peri-urban area, and Battambang province. The selection of sites was purposive, aiming to maximize the variety of participants’ views regarding NCDs, pathways to healthcare, and experiences in navigating the health system, while also taking into consideration how structural and cultural aspects impact people's health-seeking behavior. All interviews were conducted in Khmer by D.K., who is a female Cambodian researcher with a PhD in public health and long-term experience in conducting field research. The research team members did not have prior contact or pre-existing relationships with the participants. The potential participants were informed about the international nature of the study, its focus on social health protection schemes and NCDs, and the reasons for conducting it during the informed consent process. To avoid bias, the information was presented in a neutral way, without any comments that could reveal researchers’ views on the topic.

Participants were recruited using purposive sampling. Out of the 20 participants without social health protection coverage, 8 were approached directly by researchers in households and public areas (including small stores, university offices, and a temple courtyard), 6 were recruited from health centers, and another 6 from hospitals. Three FGs were conducted at health centers (one with NSSF beneficiaries and two with HEF beneficiaries), while the remaining three were held at referral hospitals (two with HEF beneficiaries and one with NSSF beneficiaries). Participants in the FGs were recruited through gatekeepers—health center workers and members of village volunteer health support groups.

Interviews lasted 30–135 min (average: 72 min), while FGs ranged from 57 to 101 min (average: 76 min). All interviews and FGs were recorded with participants’ permission. The recordings were transcribed using intelligent verbatim and then translated into English by two research assistants. Transcripts were not returned to participants for comments or corrections.

The analysis was inductive and inspired by constructive grounded theory (Charmaz 2006), an analytical framework ideal for developing comprehensive explanations grounded in empirical data. During the initial, incident-by-incident coding, the codes remained close to participants’ words, providing insights into the issues of concern for them (e.g. ‘experiencing sleep problems,’ ‘drinking lemongrass water,’ ‘using the village doctor’). After the initial coding of the entire dataset, focused coding was conducted, reviewing the emerging codes and synthesizing them into categories and sub-categories (e.g. ‘choosing traditional remedies’ and ‘barriers to seeking medical care’). Finally, the relationship between the focused codes ‘health-seeking behavior’ and ‘social health protection schemes’ was analysed to explain how access or lack of access to HEF or NSSF influences individual decisions to seek medical care for symptoms related to hypertension and diabetes. Reflective memos were used to identify potential issues of interest, document emerging patterns, elaborate categories, subcategories, and properties, and construct the arguments. The analysis was conducted using NVivo 14 software.

### Ethics

Before beginning data collection, ethical approval was obtained from both the institutional review board of the authors’ institute and the National Ethics Committee for Health Research in Cambodia. Potential participants were informed about the nature and purpose of the study, the expected duration of the interview or FG, and the rights they have as participants, including those of not answering questions they do not feel comfortable with, withdrawing from the interview without any repercussions, and asking to have their information removed from the dataset. Participants signed informed consent forms and received information sheets containing the contact information of the two co-PIs. The data were de-identified at the point of transcription.

## Results

### Lay understandings and primary prevention of diabetes and hypertension

Most participants, regardless of whether they had a chronic disease, had a general understanding of diabetes and hypertension as lifestyle-related conditions. This awareness came from a variety of sources, including doctors, diagnosed relatives, friends, and acquaintances, public health initiatives, and social media. However, their knowledge was often incomplete or inaccurate, particularly regarding causes, prevention, and management. For instance, while diet and lack of exercise were widely acknowledged as risk factors for hypertension, some participants blamed specific fruits, such as durian, or the ‘chemicals’ allegedly present in imported vegetables. Many believed diabetes was solely caused by sugar and hypertension by high salt intake. Also, several participants considered diabetes curable through biomedical treatment or traditional remedies:

‘If [one] goes to the hospital when the disease is at the early stage, it can be curable. Yet, if [one] goes to the hospital by the time the disease is getting severe, it may not be curable or it may be curable, but [they have] to spend a lot of money.’ (FG01)

‘I’ve heard on social media that there is a product of traditional medicine that can treat diabetes’ (FG06).

The primary prevention of the two conditions is informed by participants’ lay beliefs about disease causation. Dietary control and physical activity emerged as the main approaches. Many participants mentioned avoiding or drastically reducing sugary food and beverages (‘I am careful with having sugary food; I never have dessert, and I drink coffee without sugar’, IDI20), especially energy drinks, in which some found the culprit for the rise in diabetes in recent years (‘I stopped drinking [energy drinks] now. I fear my blood sugar will increase’, IDI06). Reducing the consumption of rice, Cambodia's staple food, was also widely reported (‘if we used to eat a lot of rice, we should decrease that by half’, IDI18), along with increasing vegetable intake (‘I do eat a lot of vegetables and rarely eat meat’, IDI04), limiting salt (‘If I eat salty food I may get high blood pressure’, IDI05), and cooking at home rather than eating out (‘I buy food from others for lunch, but I rarely do it in the evening. No matter how tired I am after coming back from selling, I cook my own food for dinner’, IDI17).

Many participants engaged in physical activity by doing physically demanding work (‘I just walk around in my shop to sell stuff, and I am sweating all the time’, IDI01), walking, cycling, running, and performing online workout routines (e.g. ‘I do exercise following what they did in YouTube’, IDI14).

Regular health check-ups were rarely mentioned as a preventive measure, and mainly by those with a family history of diabetes or hypertension:

“My family—my mother and father—are in their 70s, but they don't have that type of disease, just like my mother-in-law, because both of them take care of their health and maybe because it is not hereditary in our family. My husband and I have had our health checked, and we have been proven to be healthy.” (IDI11)

This reluctance to undergo regular check-ups will be further explored in the final part of the Results section. The following parts will examine the main patterns in health-seeking behavior that emerged from the study (traditional remedies, self-medication, semi-professional care, and formal healthcare), as well as the impact of social health protection on these behaviors.

### Using traditional remedies

Traditional remedies continue to be widely used for a variety of health concerns, ranging from infectious diseases like typhoid fever to the treatment of colds and cold-like symptoms, and the management of hypertension and diabetes. In the context of managing hypertension or its associated symptoms, participants reported consuming—or observing others consume—lime juice; a boiled mixture of pandan, ginger, and lemongrass; boiled lemongrass and galangal; kinin tree and moringa plant; a mixture of honey and lime juice; lemongrass water; sweetened tea; and garlic, either eaten raw or boiled. One participant mentioned a relative topically applying lime juice on the neck to lower blood pressure. Other traditional practices for addressing symptoms associated with hypertension included coin rubbing, massage, and sleeping on the ground.

Traditional healing methods are also used to manage diabetes, though they are less popular than in the case of hypertension. Some participants reported consuming a boiled mixture of ginger and lemongrass for its diuretic effect, believing it helps eliminate toxins from the body (‘we pee quite often; it releases poison’; FG02). Another participant recounted using boiled dried banana flower for a couple of years and feeling optimistic about their condition (‘I thought I would recover because I felt well after drinking that’; IDI13). Infusions of dried wild rambutan and the use of neem leaves were also mentioned as traditional diabetes remedies.

Traditional remedies are often met with skepticism in the case of diabetes because of their perceived health risks. Personal and vicarious experiences of adverse outcomes contribute to this critical view, as one participant who relied solely on biomedical treatment shared during a FG:

“[Others] got the disease later but all of them have passed away. They took both traditional and modern medicine. They had been advised to take the poison off their bodies before they could take modern medication. Those who took both are all gone. I’ve only taken medicine prescribed by the [allopathic] doctor.” (FG02)

The participant who used banana flower to lower blood sugar learned about the inefficacy of the treatment the hard way. After ceasing medication and relying solely on the herbal remedy, they started experiencing dizziness and, following a blood test, discovered that their blood sugar level was 190 mg/dl, indicative of uncontrolled diabetes.

Participants mentioned economic and cultural factors for choosing traditional healing. Traditional medicine, often plant-based, is generally cheaper than biomedical treatment, making it the first option for those in financial difficulty. As one participant mentioned, ‘I spend 200 000 riels for medication here and [those using traditional remedies] spend only 4000 to 5000 riels’ (IDI02). However, not all traditional remedies are affordable, with some concoctions costing up to 80 USD.

Cultural beliefs also shape healthcare choices. For instance, an elderly man from a well-off family (‘his children are not poor; they have at least one or two cars’; IDI14) with severe hypertension decided to leave the hospital after a day to rely on traditional medicine at home, because of his trust in Khmer medicine. Several participants also indicated that this view is not isolated: ‘[F]oreign medicine is not that better than Khmer medicine, they are the same’ (IDI09); ‘If we have some food that is good for health, like honey, we don't need to go see the doctor’ (IDI11). While many acknowledged biomedicine's effectiveness, some viewed it with caution, as ‘a two-faced weapon. It can treat your illness, but it also affects some [other] part of your body’ (FG06). By comparison, traditional medicine is not generally believed to have major side effects. Social factors also influence the use of traditional remedies. Community discussions, stories of healing or worsening health, and the sharing of concoction recipes for different conditions shape individual choices. However, based on participants’ accounts, younger people are generally less inclined to use traditional remedies as compared to older ones, but more open to the neotraditional products promoted on social media, including ones claiming to ‘cure’ diabetes.

### Self-medicating

Many participants either replace or complement traditional healing methods with medications purchased from local pharmacies or other drug-selling facilities without a prescription or medical guidance. Self-medication is common for minor ailments, such as headaches, dizziness, fatigue, swelling of the hands or legs, or burning feet (‘my feet burn like the candle,’ FG02). A typical pharmacy visit follows this pattern:

“I go to the pharmacy and tell them I feel dizzy and not feeling well. Then they ask if I have heart trembles or not, suffocation or not, because I am fat. And I say I do not suffocate, but I don't feel well. So, they combine this and that. I am not really sure because I haven't been to a place that does blood tests or health check-ups. […] Like, when I have a headache, they ask me how many days I want medication for; they ask if I have a cough. If I only have a headache, they only give medication for the headache, and then I take that. It's not really a clear diagnosis from a doctor. It's not like when we go to a place that checks our health” (IDI12).

Others go to pharmacies already knowing what they want—either refilling expired prescriptions, repurchasing medication that previously worked for them, or obtaining drugs that helped someone else with similar symptoms. To ensure they receive the right product, many bring an empty package of the desired drug with them. Pharmacies often dispense these medications freely, including those requiring a prescription, like antibiotics.

As with traditional remedies, cost is the primary reason for choosing a pharmacy as the first point of contact for healthcare: ‘If I go to the pharmacy, I spend only 20 000 riels [5 dollars], [and] I can go home. If I go to the hospital, I need to have 100 dollars or 200 dollars in my pocket’ (IDI12). Expensive private care discourages even those who trust biomedicine, while public health centers, despite giving free services to HEF and NSSF beneficiaries and having lower costs than other health facilities, are seen as unappealing due to long wait times, staff shortages, medication unavailability, and a perceived low quality of care.

Beyond affordability, pharmacies are also popular because of their accessibility and convenience. They are widely available in urban and rural areas, with some operating outside regular hours. For instance, one participant (IDI09) noted the existence of six or seven pharmacies or drug stores in his village, some of which are conveniently located near the market. A participant for whom pharmacies are the first choice for seeking care shared her views of the tradeoff between speed and medical oversight:

“Yes, they don't check on me [at the pharmacy]. I just tell them about my condition, and they give me the medicine. The medical staff at the health center give me examination, but I have to wait for a long time.” (IDI08)

Despite the widespread trust in pharmacies, several participants criticized their insufficient regulation and oversight. Concerns included unqualified workers (‘In our country, those who want to open a pharmacy open one, but they don't seem to have expertise in this’, IDI11) and pharmacists prioritizing profit over safety by selling counterfeit or substandard medicines (‘Sometimes, […] they sell medicine with no specific manufacture, source, and license’, IDI10).

### Seeking care in the semi-professional sector

Pharmacies and drug stores not only dispense medications but many of them also offer blood pressure checks and blood glucose tests. While these services are not free, they remain affordable for most individuals (for instance, a blood sugar test costs about 5000 riels; FG03). However, the interpretation of results varies widely from one pharmacy to another. In some cases, test results directly determine medication, with pharmacists substituting for doctors in diagnosing and prescribing treatment. One participant criticized this practice, arguing that pharmacies provide suboptimal and unstandardized care, potentially endangering patients:

“Let's say you go to the pharmacy. You can ask them to do a blood test. However, it is not as specific as the blood test in the hospital. At the pharmacy, they will tell you the result like ‘Oh, your blood sugar has increased to this level,’ and they will give you medicines. Later, if you are not feeling well, your blood sugar increases, and you are going to the pharmacy again, they will give you another type of medicine. If you go to the hospital, the medical professionals will consult with you and give you advice on what to do, what to eat or exercise. At the pharmacy, they will give you any medicines according to your symptoms, no matter if your blood sugar level is high or low.” (FG04)

However, some pharmacies act as a referral point rather than an alternative to professional care. Several participants reported that when their blood pressure or glucose levels tested high, pharmacy workers recommended seeking further evaluation at a health center, public hospital, or private practitioner: ‘After they tested my blood, they told me my blood pressure wasn't good and I should go to meet the doctor at the hospital’ (IDI02). Thus, pharmacies sometimes serve as an entry point into the broader healthcare system for individuals who might have otherwise delayed seeking care.

Village healers often play a similar role, though they typically do not perform tests themselves. Instead, they rely on their experience to recognize symptoms and advise patients to go for biomedical evaluation. For instance, one participant seeking intravenous therapy for symptom relief, a common practice in Cambodia (Ovesen and Trankell 2016, Verschuere *et al.* 2017), was urged by a village doctor to undergo further testing:

“[T]he doctor I asked to come to my house suspected that I have diabetes because I suddenly lost a lot of weight. So, the doctor told me to go to the private provider and have my blood tested. I did as what the doctor instructed and found out that my blood sugar level had increased to 220 [mg/dl]” (IDI14).

The cases discussed above indicate that the semi-professional sector—pharmacies and village healers—often acts as a bridge between lay and specialized care, encouraging individuals with concerning symptoms and signs to seek medical evaluation and diagnosis. However, the process of referring patients is unregulated, *ad hoc*, and dependent on the goodwill of pharmacy workers and village healers.

### Seeking care in the professional sector

In many cases, significant delays occur between the onset of symptoms and seeking biomedical care. One participant (ID15) shared his experience, which is common among individuals diagnosed with diabetes. He first noticed symptoms, including weight loss, nearly 20 years earlier, in 2004, but dismissed them as they did not affect his work. As he continued to lose weight, colleagues and acquaintances expressed concerns that something was amiss. Initially suspecting HIV rather than diabetes, he avoided testing. It was only after experiencing severe back pain that he sought care at a private medical practice, where he stayed overnight. During his stay, he noticed frequent urination and mentioned it to the practitioner, who advised a blood test at a nearby clinic. The results revealed a staggering blood sugar level of 370 mg/dl.

This tendency to overlook initial symptoms is not uncommon. Many individuals rationalize the symptoms they experience, attributing them to daily work rather than potential illness. As one FG participant commented,

“[E]lderly people or those who live in the province always think that if they worked hard, that's why they feel dizzy, or if they haven't eaten anything, that's why they feel dizzy. They never think they might have a severe illness. They wait until they collapse, and only then they agree to go to the hospital.” (FG05)

While poor health awareness or complacency may contribute to this reluctance, as alluded to by the participant above, focusing solely on individual shortcomings overlooks broader structural factors that shape people's decision-making. Fear plays a major role in deterring individuals from seeking medical help. Several participants described avoiding hospitals due to fear of diagnosis: ‘[My mother] said that she wouldn't go [to the hospital] because if she found out she has a lot of symptoms, she would be very afraid,’ FG03; ‘Some people are afraid that they really have diabetes so they don't go to see the doctor,’ FG03. This fear is sometimes due to the fatalistic belief that diabetes is the end of the road (‘[They are] afraid that [diabetes] is not a treatable disease’), a view echoed by several participants. However, for many others, the fear is primarily related to the economic burden that biomedical care would impose on the patient and their family (‘I’m afraid too before going to hospital. I don't believe in the doctor. First of all, it's costly,’ FG02).

Several participants discussed the hesitancy to seek care at the intersection of poverty and the cultural norm of not burdening others, especially family members: ‘There are some people who think like that: if they go to the hospital, they will spend money, so they won't have money to support the family in the everyday life. So, they don't go to the hospital’ (FG05). This line of reasoning is predominantly attributed to males, who are traditionally expected to be the breadwinners in Cambodian society: ‘[T]he head of the family […] needs to make money. In general, the head of the family is strict in spending money’ (FG05). However, examples of females refusing to go to the doctor due to concerns about the financial well-being of the family suggest that this orientation spans the gender divide: ‘For instance, my mom, she doesn't like going to the hospital when she gets sick because she is concerned that I have to spend too much money on her medical expenses’ (FG06).

### Social health protection and health-seeking behavior

A significant barrier to seeking preventive care is financial. One participant's (IDI10) narrative was particularly telling of the relation between social health protection schemes and his approach to health care. Initially, he worked for a brief period with an international NGO that offered him private insurance linked to a specific health provider. Later, he joined an international company that provided him with benefits under the NSSF. Motivated to monitor his health condition, he utilized this insurance for regular check-ups, either monthly or bimonthly. He sometimes visited reputable public hospitals, such as Calmette and the Khmer–Soviet Friendship Hospital, informally known as the Russian Hospital (Grant 2018), but more often used a private facility contracted with the NSSF because of its perceived high-quality service and much shorter wait times. After 15 years with that company, he had to resign when it relocated far from the capital, making it impractical for him and his family to move there or commute daily. After that, like many other urban Cambodians, he ventured into the informal economy. Initially, he worked as a beer seller, and then as a tuk-tuk driver, occupations that did not come with any social health protection. Faced with the prohibitive cost of check-up packages (∼$180), he reluctantly ceased routine examinations despite being concerned for his health.

The dependence on social health protection to afford medical care is not unique to preventive measures but also extends to seeking assistance after experiencing distressing symptoms. Another participant without health insurance succinctly described a widespread decision-making process: ‘Every person wants to live. If we have an ID Poor card or an NSSF card, we will go to the hospital. Without these cards and with no support, we won't go and will endure the pain’ (IDI14). As previously noted, most individuals who cannot afford a doctor's visit do not passively await the worsening of their condition. Instead, they often turn to folk remedies and self-medication at the beginning of symptoms, resorting to pharmacies and village healers if these measures do not yield the expected benefits. However, the key idea that for many facing severe economic hardship, social health protection is instrumental in seeking care has been echoed by numerous participants.

In the wake of this observation, it is important to acknowledge that while having HEF or NSSF coverage is necessary, it often proves insufficient for accessing quality healthcare services. Structural challenges within the healthcare system often undermine the benefits offered by these social health protection schemes. A notable example is the limited availability of diabetes services at health centers, which pushes HEF beneficiaries to look for care at referral or provincial hospitals or use the services of a local NGO. These options come with a significant financial burden, as the monthly cost of insulin injections and medication to be paid out-of-pocket could be as high as 160 000 riels (participant, FG02). Moreover, the distance to the hospitals, the difficulty in finding transportation, and the time lost with seeking care are important barriers for seeking care: ‘First is money, second is the time of person who accompany her to hospital, third is the transportation […] They will waste one day if they go to the hospital,’ FGD05.

The situation is more favorable in the case of hypertension, as health centers conduct screenings and offer treatment on the premises. Nevertheless, the medication provided in public health facilities usually covers only a week to a month, requiring frequent returns for prescription refills. In the context of time lost to transportation and protracted waiting, combined with the risk of medicines being out of stock, many HEF beneficiaries decide to purchase their treatments from pharmacies, incurring additional out-of-pocket expenses.

## Discussion

This study examined health-seeking behaviors related to hypertension and diabetes in Cambodia and the impact of social health protection on decision-making. The findings indicate that participants generally recognize these diseases as serious, lifestyle-related, and on the rise in Cambodia, and correctly identify their most common symptoms. The perceived susceptibility and severity often translate into action, many participants reporting dietary changes and increased physical activity as ways to reduce their risks. However, primary prevention is weakened by misconceptions, such as focusing exclusively on sugar, salt, and specific foods like durian, while overlooking other risk factors, like total calorie intake.

This situation changes in tertiary prevention. Individuals who have been diagnosed appear more inclined to follow medical advice rather than information from other sources. They seem more committed to lifestyle changes and treatment adherence, with many combining traditional Khmer remedies with medical care. This finding is consistent with an earlier study conducted in a diasporic Cambodian community (Renfrew *et al.* 2013). However, participants have also reported cases of using traditional remedies as alternative treatment, particularly in managing hypertension. Of particular concern is the growing popularity of neotraditional products (i.e. commercially produced remedies that draw on the principles and ingredients of traditional medicine) that falsely claim to cure diabetes. A similar phenomenon was documented in Ghana (Abdulai *et al.* 2022). Fear of side effects and misunderstanding the need for continuous treatment make such products appealing, potentially leading to delayed care and increased risk of complications.

Secondary prevention appears to remain a major challenge. Although participants tend to recognize disease severity, screenings are rare, possibly due to the insufficient awareness of the asymptomatic nature of hypertension and early-stage diabetes. Fear of diagnosis, sometimes related to fatalistic beliefs, also discourages proactive screening. However, opportunistic blood pressure checks at health centers and pharmacies contribute to the early detection of hypertension.

Previous work has identified significant delays between disease onset and diagnosis for both hypertension and diabetes (Jacobs *et al.* 2017, Te *et al.* 2023), a finding confirmed by our study. In addition to insufficient screenings, many individuals experiencing typical symptoms dismiss or rationalize them (e.g. attributing them to intense work), initially relying on herbal medicine, traditional healing practices, and self-medication. When these measures fail, they often turn to the semi-professional sector, represented by village healers and pharmacy workers. This pattern is common in many low- and middle-income countries (LMICs) with fragmented health systems and high out-of-pocket healthcare costs, such as India (Yellapa *et al.* 2017) and Bangladesh (Jennings *et al.* 2021).

Direct and indirect costs associated with healthcare emerged as the most significant deterrent to seeking professional care, a finding consistent with previous studies (Khun and Manderson 2008, Nang *et al.* 2019, Kolesar 2023). The social norm of minimizing the burden on family members was also widely mentioned, likely reflecting past experiences of catastrophic health expenditure (Damme *et al.* 2003). Faced with high costs for consultation, laboratory tests, procedures, and medication, as well as informal payments (Page *et al.* 2020), many people in dire economic situations resorted to borrowing from friends, relatives, or microfinance institutions, or selling assets such as land or livestock to cover these costs (Kolesar *et al.* 2023). Such actions pushed many into poverty or exacerbated their poverty status.

The expansion of social health protection schemes (HEF and NSSF) has reduced financial barriers and improved access to care. Indirectly, HEF has also increased hypertension screening, as blood pressure checks are routinely conducted during consultations at most health centers. Participants’ accounts of changing health-seeking behaviors after gaining access to social health protection indicate their role in prevention, early diagnosis, and treatment of health problems (see also Treleaven and Ngin 2021). However, in the case of NCDs, the benefits of HEF are limited by: structural problems in primary care, such as the lack of diabetes services, including screening, at most health centers (Chhim *et al.* 2023); frequent medication stockouts (Jacobs *et al.* 2017); prescriptions for a short period (Nang *et al.* 2019); and long waiting times (Verschuere *et al.* 2017). These problems drive many HEF beneficiaries to seek private care or buy medicines directly from pharmacies, a pattern also encountered in other LMICs (Chary *et al.* 2023). NSSF beneficiaries fare better, as they can access both public and private providers, reducing out-of-pocket spending. Therefore, strengthening primary care, expanding diabetes services at public health centers, and extending the coverage of social health protection schemes are necessary to prevent worsening health inequities.

Previous work has argued for expanding the role of non-medical professionals, such as the peer educators, who are part of a network developed by the local organization MoPoTsyo (Steinman *et al.* 2020), and community health workers (Chham *et al.* 2024), in the management of NCDs. In neighboring Vietnam, village health workers have recently been mobilized to conduct blood pressure and glucose testing (Long *et al.* 2020). Our study recommends considering another overlooked group-pharmacy workers and village healers—who already contribute to early detection of hypertension and diabetes through opportunistic testing and referral to health centers. However, these activities are inconsistent and contingent upon testing equipment and experiential knowledge.

Since the semi-professional sector enjoys credibility and trust in Cambodia (Ovesen and Trankell 2016), providing village healers and pharmacy workers with training and basic equipment (e.g. blood pressure cuffs and glucose meters), reimbursing expenses, and incentivizing successful referrals could contribute to the early detection of the two conditions. Formalizing their role in secondary prevention could improve diagnosis rates, reduce complications, and lower overall health costs. In this respect, our study is consistent with calls from other LMICs (Khanal *et al.* 2016, Correr *et al.* 2020, Jonkman *et al.* 2020, Sousa Pinto *et al.* 2020) to recognize the potential contribution of pharmacists and pharmacy workers in the management of NCDs. Accredited traditional medicine practitioners could also play a complementary role in NCD management, especially given the widespread use of traditional remedies reported by patients. At the same time, nutrition specialists and health promoters could support the prevention and self-management of chronic conditions by increasing nutrition literacy and encouraging dietary changes.

### Limitations of the study

Since the FGs and many of the interviews were conducted at health centers or referral hospitals, it is likely that individuals who are more familiar with or have better access to formal healthcare services are overrepresented in the sample. Their views and experiences may differ from those of more marginalized individuals, and those who avoid or distrust public healthcare. This potential bias limits the generalizability of the findings and calls for additional studies using community-based sampling approaches.

## Conclusions

As Cambodia progresses through an epidemiological transition, addressing the growing burden of NCDs presents an important challenge for its health system. Our findings indicate that participants have some awareness of and concern about hypertension and diabetes, recognizing that these conditions are considerably more prevalent now than in the past. This awareness often leads to the adoption of healthier behaviors, such as reducing the intake of salt or carbohydrates and engaging in physical activity. However, the understanding of risk factors and prevention is uneven and often marred by misconceptions, which indicates that health education needs to be strengthened.

Secondary prevention continues to be a major concern, as many people do not undergo routine screenings for hypertension and diabetes, do not have regular check-ups, and delay seeking biomedical care even after experiencing concerning symptoms. Instead, many rely on self-care, pharmacies that offer testing and guidance, or village healers.

Financial constraints remain an important barrier to preventive and ongoing care, making social health protection schemes such as HEF and NSSF necessary for accessing biomedical services. However, while these programs help reduce financial burdens, they are often insufficient in ensuring comprehensive care, especially for diabetes. The absence of diabetes services at many health centers affects secondary and tertiary prevention, increases direct and indirect costs for patients, and reduces treatment adherence. The management of hypertension fares better, but frequent medication shortages and the need for regular prescriptions drive many individuals, especially those without social health protection, to turn to self-care, traditional remedies, and pharmacy-based care.

Our findings support the need for a more integrated health system approach, enhancing the capacity of health centers to manage NCDs effectively. This includes expanding the availability of essential medicines and services, adjusting reimbursement thresholds to allow doctors to provide longer treatment periods, and finding solutions to reduce waiting times. Furthermore, taking advantage of the human resources in the semi-professional sector, particularly pharmacy workers and village healers, could be instrumental in the early detection and referral of cases of diabetes and hypertension to appropriate healthcare facilities.

## Data Availability

The data that support the findings of this study are available from the corresponding author upon reasonable request.

## Reflexivity statement

This paper is part of a larger collaborative effort between the Global Health Research Center at Duke Kunshan University (China) and the University of Health Sciences in Phnom Penh (Cambodia), initiated in 2023 through the signing of a Memorandum of Understanding between the two institutions. The team includes an equal number of researchers (2) from each collaborating institution. Also, two authors (the first author and the corresponding author) are early-career researchers, whereas the other two authors are senior researchers. The corresponding author is female, while the other three authors are male.
